# Presence of stromal cells in a bioengineered tumor microenvironment alters glioblastoma migration and response to STAT3 inhibition

**DOI:** 10.1371/journal.pone.0194183

**Published:** 2018-03-22

**Authors:** R. Marisol Herrera-Perez, Sherry L. Voytik-Harbin, Jann N. Sarkaria, Karen E. Pollok, Melissa L. Fishel, Jenna L. Rickus

**Affiliations:** 1 Department of Agricultural and Biological Engineering, College of Engineering, Purdue University, West Lafayette, Indiana, United States of America; 2 Physiological Sensing Facility at the Bindley Bioscience Center and the Birck Nanotechnology Center, Purdue University, West Lafayette, Indiana, United States of America; 3 Weldon School of Biomedical Engineering, College of Engineering, Purdue University, West Lafayette, Indiana, United States of America; 4 Department of Basic Medical Sciences, College of Veterinary Medicine, Purdue University, West Lafayette, Indiana, United States of America; 5 Department of Radiation Oncology, Mayo Clinic, Rochester, Minnesota, United States of America; 6 Indiana University School of Medicine, Department of Pediatrics, Wells Center for Pediatric Research, Indianapolis, Indiana, United States of America; 7 Indiana University School of Medicine, Department of Pharmacology and Toxicology, Indianapolis, Indiana, United States of America; 8 Indiana University Simon Cancer Center, Indianapolis, Indiana, United States of America; Northwestern University, UNITED STATES

## Abstract

Despite the increasingly recognized importance of the tumor microenvironment (TME) as a regulator of tumor progression, only few *in vitro* models have been developed to systematically study the effects of TME on tumor behavior in a controlled manner. Here we developed a three-dimensional (3D) *in vitro* model that recapitulates the physical and compositional characteristics of Glioblastoma (GBM) extracellular matrix (ECM) and incorporates brain stromal cells such as astrocytes and endothelial cell precursors. The model was used to evaluate the effect of TME components on migration and survival of various patient-derived GBM cell lines (GBM10, GBM43 and GBAM1) in the context of STAT3 inhibition. Migration analysis of GBM within the 3D *in vitro* model demonstrated that the presence of astrocytes significantly increases the migration of GBM, while presence of endothelial precursors has varied effects on the migration of different GBM cell lines. Given the role of the tumor microenvironment as a regulator of STAT3 activity, we tested the effect of the STAT3 inhibitor SH-4-54 on GBM migration and survival. SH-4-54 inhibited STAT3 activity and reduced 3D migration and survival of GBM43 but had no effect on GBM10. SH-4-54 treatment drastically reduced the viability of the stem-like line GBAM1 in liquid culture, but its effect lessened in presence of a 3D ECM and stromal cells. Our results highlight the interplay between the ECM and stromal cells in the microenvironment with the cancer cells and indicate that the impact of these relationships may differ for GBM cells of varying genetic and clinical histories.

## Introduction

Glioblastoma (GBM), the deadliest type of brain cancer[1], establishes a synergistic relationship with its local environment to support tumor growth, migration, and therapy resistance. These interactions lead to the formation of the tumor microenvironment (TME), which is comprised of supportive stromal cells and surrounding extracellular matrix (ECM)[2–6]. Despite the increasingly recognized importance of the TME as a modulator of GBM progression, our understanding of its specific role on processes such as migration or survival has been challenging given the complexity and reciprocity of the TME interactions.

Glioblastoma cells remodel the normal brain microenvironment and in turn this altered microenvironment supports tumor growth. GBM cells directly deposit proteins such as fibrillar collagen[7,8] and fibronectin[9], naturally absent in normal brain ECM[10,11], presumably to increase tissue stiffness and facilitate cancer migration. Stromal cells are recruited to the TME to support tumor growth, invasion, and hinder immune surveillance[3,12,13]. Endothelial cells are attracted by proangiogenic signals to form new vascular networks[14]. The new vasculature provides oxygen and nutrients to the tumor, and indirectly serves as migration routes[11]. Paracrine signaling between GBM and astrocytes has also been shown to induce astrogliosis in a process that recapitulates the astrocytic response to brain injury[15]. The secretion of pro-inflammatory signals by astrocytes promotes GBM migration, proliferation, and also acts as a protection barrier for immune T-cell infiltration[12,13,16].

The altered tumor microenvironment not only modulates gliomagenesis but also GBM therapy response. Previous studies have shown that properties of a three-dimensional (3D) ECM[17–19] and presence of stromal cells such as astrocytes and microglia[20,21] influence tumor survival after drug treatment (when compared to standard liquid monoculture - 2D culture). Such studies combined with the poor translation rate of therapies from the lab to the clinic, suggest that new laboratory models that better represent the human brain are needed.

Although multiple chemotherapeutic targets have been evaluated for GBM, advances in medical treatments have shown only minimal improvement in GBM patient’s survival. Less than 5% of GBM patients reach a 5-year survival milestone[1]. STAT3, a member of the Signal Transducer and Activator of Transcription (STAT) family, has attracted wide attention as a target for GBM treatment given its role in multiple cellular processes including proliferation, survival, and migration[22,23]. STAT3 activity is directly regulated by signals from the microenvironment, including various growth factors (EGF, bFGF, VEGF) and cytokines, which are abnormally expressed by GBM[24]. STAT3 has been found constitutively active in 9 to 83% of human GBM tumors[23] and its activity has been linked to increased migration through regulation of cell adhesion mediated by Rho GTPases, and higher expression of matrix metalloproteinases (MMPs)[25,26].

In order to understand the complex role of the TME as a modulator of GBM behavior new *in vitro* models should represent the TME components, including stromal cells and the 3D ECM. Multiple studies have focused on the GBM-ECM[27–31] or GBM-stromal cell interactions using 2D platforms[12,13,20,32], however, few models have been developed to study the role of stromal cells on cancer in a 3D ECM context[33,34]. *In vitro* models comprised of multiple distinct cell types and 3D matrix are powerful tools that can be tuned to represent the characteristics of the GBM TME in a controlled and systematic manner[31]. These models offer a more realistic approach to study intra- and inter-cellular signaling in the TME and evaluate possible drug targets, such as STAT3.

Here, we developed a 3D *in vitro* model that exhibits characteristics of the GBM TME, including low stiffness, hyaluronan composition, and presence of stromal cells, to evaluate their effect on GBM migration and on survival and migration after anti-STAT3 treatment. To recapitulate the physical and compositional characteristics of GBM ECM we used a previously developed 3D matrix comprised of standardized oligomer type-I collagen and hyaluronan that presents low stiffness[31]. In addition, stromal cells such as human astrocytes, the main non-neuronal stromal cell type of the brain[15] and human endothelial colony forming cells (ECFCs) were incorporated into the matrix and cultured with patient-derived GBM cell lines (GBM10 and GBM43) and the stem-like GBM line GBAM1 (CD133+). ECFCs are endothelial cell precursors able to undergo vasculogenesis when cultured or transplanted in 3D collagen matrices[35,36]. In addition, ECFCs spontaneously form human blood vessels that inosculate upon *in vivo* implantation[37–39]. The GBM TME 3D model was used to study the effect of 3D ECM and stromal cells on GBM migration characteristics (total distance, net displacement and directionality of movement) and on survival and migration following treatment with the small-molecule STAT3 inhibitor SH-4-54[40,41].

## Materials and methods

### Standard liquid cell culture

GBM human–derived cell lines GBM10 (recurrent GBM; wildtype p53, CDKN2A deletion, wildtype STAT3) and GBM43 (primary GBM; mtp53, CDKN2A deletion, wildtype STAT3) were maintained in high-glucose DMEM (Life Technologies, Carlsbad, CA) supplemented with 10% FBS as has been described previously[42–44]. GBM human cell line GBAM1 (neurosphere forming, self-renewal, CD133+, SOX2+, Notch+, GFAP-) was originally developed by Dr. Philip Tofilon and the Moffitt Cancer Center from GBM surgical specimens, sorted for CD133+ (final population CD133+>90%) and tested for continuous self-renewal, differentiation to glial and neuronal precursors and tumor formation in nu/nu mice[45]. GBAM1 was maintained in DMEM/F12 supplemented with B27 without vitamin A (Life Technologies, Carlsbad, CA) and with growth factors EGF, bFGF (50 ng/ml each, Peprotech, Rocky hill, NJ). Human primary astrocytes from ScienCell (Carlsbad, CA) were maintained according to vendor specifications. Endothelial umbilical cord blood ECFCs, kindly provided by Dr. Mervin Yoder (Indiana University School of Medicine), were maintained in collagen type-I coated plates with EGM-2 medium (Lonza, Walkersville, MD) as described by Whittington (2013)[35]. All cell lines were cultured at 37°C in an atmosphere of 5% CO_2_, fed with complete media every other day and passaged at 70–80% confluence. To obtain astrocyte conditioned medium, astrocytes were seeded at an initial density of 5000 cell/cm^2^ and cultured according to vendor specifications for 5 days, the media was collected centrifuged at 1000 rpm for 5 min to eliminate possible present cells. Cells used to evaluate STAT3 activation after IL-6 treatment were cultured for 36 h (~80% confluency) and stimulated with 30 ng/ml of IL-6 (Cell Signaling Technologies, Danvers, MA) for 30 min before recovery for protein extraction.

### Synthesis of 3D brain-like matrix and 3D cell culture

3D matrices of collagen-hyaluronan were prepared by polymerizing standardized type I oligomer collagen[46] (2 mg/ml) and sodium hyaluronate (10 mg/ml; MW 351–600 KDa) (Lifecore Biomedical, Chaska, MN) as previously described[31]. Cells were suspended within collagen-hyaluronan solution at specified densities prior to polymerization at 37°C for 30 minutes. Complete media was added to the top of the matrix, and cells were cultured at 37°C in an atmosphere of 5% CO_2_.

### Migration in 3D brain-like matrix

GBM cells were stained with CellTracker™ Green CMFDA dye (Life Technologies, Carlsbad, CA) and embedded at a density of 1*10^6^ cells/ml within the collagen-hyaluronan matrix before polymerization. For assays involving astrocytes and/or ECFCs, all the cells were embedded at the same time in the matrix prior polymerization and at the same density (1*10^6^ cells/ml each population). Volumes of 30 μl of matrix per well were platted in a μ-slide angiogenesis chamber (Ibidi, Germany). After matrix polymerization, 30 μl of media were added per well, co-cultured cells were fed with media containing equal volumes of GBM, astrocytes and ECFC media and maintained in incubation at 37°C in an atmosphere of 5% CO_2_ for 24 h. Afterwards, cells were placed in an on-stage incubator to perform time-lapse confocal microscopy every 90 min during 15 h.

### Time-lapse confocal imaging and migration analysis

Cell migration was monitored by time-lapse microscopy using an Olympus FV1000 confocal microscope. Optimal growth cell conditions were maintained using on-stage incubator chamber at 37°C in an atmosphere of 5% CO_2_. Z-stacks of 200 μm were acquired using 12–15 μm steps; initial and final z positions were chosen to be at least 50μm separated of the surface or the plate interface. Different areas (4 to 9 areas) were acquired per sample (each individual area of 0.0187 mm^2^) to cover at least 60% of the total area of the well. Image stacks were projected as XY images for migration analysis. Trackmate plugin from FIJI was used to analyze the time-lapse images using LoG (Laplacian of Gaussian) detector, assuming a blob diameter of 10 pixels (all images were 512 pixels, 2.67 μm per pixel) and threshold of 1 pixel, without sub-pixel localization. LAP tracker option was chosen allowing frame to frame linking and closing of 15 pixels in 3D migration experiments and 25 pixels in 2D migration experiments. Data was filtered to only account for cells visible during the total time of the experiment. Raw data from Trackmate was analyzed using the Chemotaxis tool plugin for ImageJ (Ibidi, Germany) to obtain total migration distance, net displacement and directionality (ratio of net displacement to total migration distance).

### Modified 3D co-culture culture method for protein extraction

GBM cells were embedded in collagen-hyaluronan matrices at a density of 3.5*10^5^ cells in 200 μl of matrix and platted in 48 multi-well plates for polymerization. To achieve 3D co-culture of astrocytes and GBM that allowed protein extraction of the different populations, the polymerized matrix containing the GBM cells was recovered and placed in the center of a well in a 24 multi-well plate. Subsequently 200 μl of collagen matrix with 3.5*10^5^ astrocytes were pipetted to the surroundings to form a concentric ring with the astrocytes layer in the outside and the GBM layer inside. The matrices were incubated during 30 min at 37°C to allow complete polymerization. Media containing equal amounts of astrocyte and GBM media were added to the culture. Cells were maintained at 37°C in an atmosphere of 5% CO_2_ for 72 h. For protein extraction, the concentric ring was separated to obtain the layers containing each of the cell populations.

### Western blot analysis

Cells cultures were washed with ice-cold PBS and incubated with RIPA buffer supplemented with 1X Halt™ protease and phosphatase inhibitor cocktail (ThermoFisher Scientific, Waltham, MA) at 4°C for 5 min (2D liquid culture) or 4 h (3D culture) with constant agitation followed by centrifugation at 12000 rpm during 20 min to recover the supernatant. Total protein concentration was quantified by Pierce BCA protein assay (ThermoFisher Scientific, Waltham, MA). Equal amounts of protein samples were denaturalized and loaded in 4–20% polyacrylamide gels (Biorad, Hercules, CA). Samples were transfered to PVDF membranes (ThermoFisher Scientific, Waltham, MA). Membranes were blocked with Odyssey blocking solution TBS (Licor, Cambridge, UK) for 1 h. Blotting of the membranes was done with primary antibodies against STAT3-p705 (dilution 1:2000), STAT3 (1:1000), β-actin (1:1000) (Cell Signaling, Danvers, MA). Secondary antibodies IRDye800CW anti-rabbit and IRDye680RD anti-mouse (Licor, Cambridge, UK) were used at a dilution 1:2000. Visualization was performed in Odyssey Clx System (Licor, Cambridge, UK). Semiquantitative analysis of STAT3 activation was done in ImageJ. Bands corresponding to phosphorylated STAT3 and total STAT3 were normalized to a ladder band to avoid differences in multiple readings of the same membrane. STAT3 activation was quantified as phosphorylated STAT3 over total STAT3.

### Drug treatment and cell viability analysis

GBM cells were cultured for 24 h (in liquid medium or within the 3D Col-HA matrix) in a 96-well plate at 5000 cells/well with 100 μl of complete media prior addition of SH-4-54 inhibitor in DMSO vehicle. Viability of GBM cells cultured in liquid media and 3D collagen-hyaluronan matrix was assessed 72 h after drug treatment by Alamar blue assay (ThermoFisher Scientific, Waltham, MA). Cells were incubated with Alamar blue reagent during 3 h and absorbance was measured at 570 nm. Based on previous measurements of diffusion in collagen gels by Ramanujan et al (2002)[47], we estimated a diffusion coefficient of 300 μm^2^/s and approximately 3 h for complete drug diffusion throughout the matrix (similar time for Alamar blue compound). Cells treated only with DMSO served as controls. For viability assays involving co-culture of GBM with astrocytes and ECFCs, the GBM cells were labelled with Cell Tracker Green CFMDA (ThermoFisher Scientific, Waltham, MA) before 3D culture. 72 h after drug treatment the 3D co-cultures were treated with eFlour 660 dye (Affymetrix, Santa Clara, CA) for 10 min. Confocal microscopy was performed to co-localize cells labeled with both dyes and quantify GBM viability.

### RNA interfering assays

GBM43 and GBM10 were transfected by reverse transfection with STAT3 siRNA (Santa Cruz Biotechnology, Dallas, TX). Briefly, 10 pmol of siRNA were diluted in 50 μl of OptiMEM medium (ThermoFisher Scientific, Waltham, MA) and mixed with 3 μl of Lipofectamine RNAimax (ThermoFisher Scientific, Waltham, MA) in 50 μl of OptiMEM media. The mix was incubated at room temperature for 15 min and added to a well of a 24-well plate. Then, 1*10^5^ GBM10 or GBM43 cells suspended in OptiMEM media were deposited per well containing the mix to a final volume of 300 μl/well. Cells were incubated at 37°C in an atmosphere of 5% CO_2_ during 24 h and 300 μl of normal DMEM media with 20% FBS were added to each well to stimulate cell attachment to the plate. Forty-eight hours after transfection cells were recovered by trypsin exposure and cultured accordingly for the migration assays or for protein recovery. Negative transfection control was transfected with control-siRNA A and positive control with siRNA FITC Conjugate-A (Santa Cruz Biotechnology, Dallas, TX).

### Statistical analysis

Data is presented as boxes indicating first, second and third quartile, outliers are presented as red dots. Other measurements are expressed as mean ± SE. Comparisons between treatments were made using two samples t-test, Kruskal-Wallis test (non-normal variance) with Tukey-Kramer mean comparison. For GBM migration in presence of ECFC and ECFC-Astrocytes the data correspond to three non-independent replicates. All other tests correspond to statistically independent repetitions (n> = 2). Comparison between groups was done by pooling all data Statistical significance was evaluated at α = 0.05.

## Results

**Fig 1:**
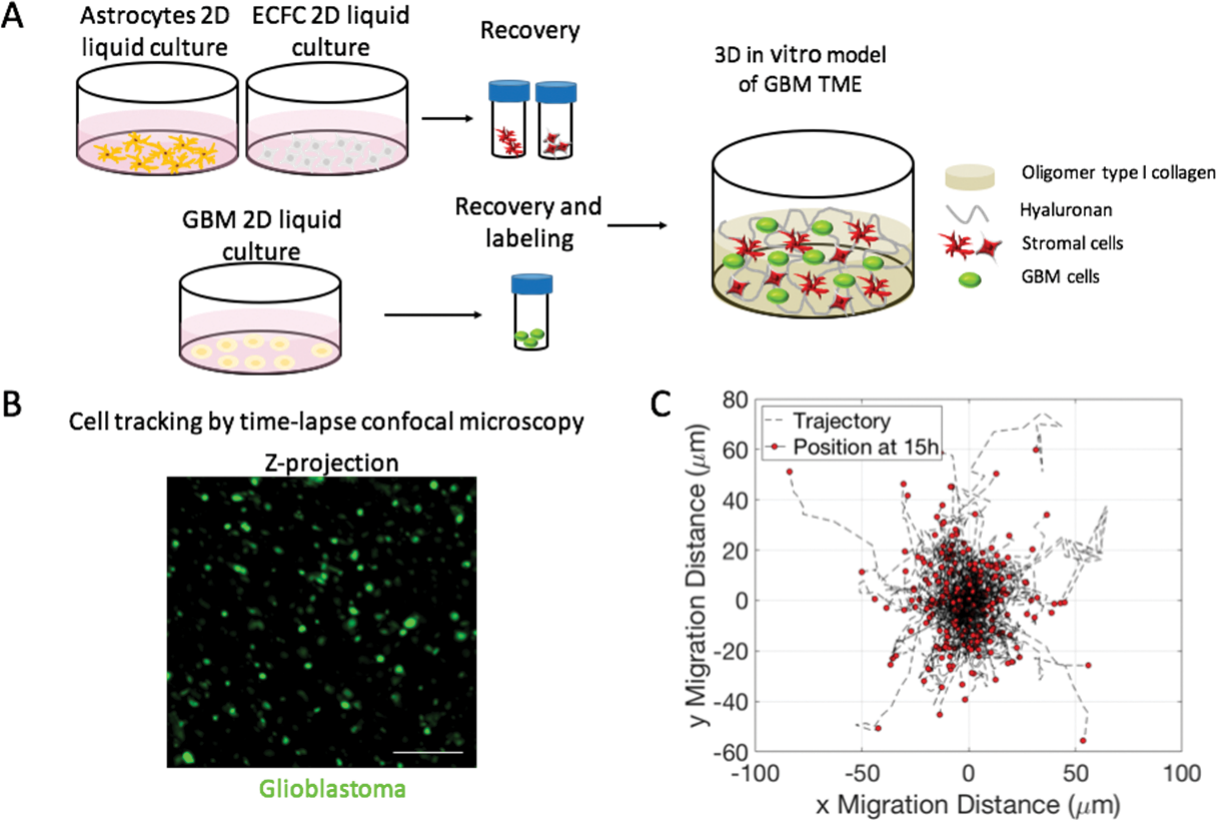
Experimental setup to analyze GBM migration in 3D co-culture with stromal cells. (A). Cell populations are expanded in 2D liquid culture, recovered, fluorescently labeled and homogenously encapsulated within a 3D matrix formed by polymerization of an oligomer collagen-hyaluronan solution. (B). The position of individual glioblastoma cells (green) is tracked by confocal microscopy every 1.5 h during 15 h. (C). The trajectories of GBM single cells are analyzed to obtain accumulated migration distance, net displacement, migration velocity and directionality. Z-stack: 200 μM, step: 12 μM. Bar 100 μM.

The GBM TME model here starts with the previously developed collagen I / hyaluronan matrix with a low elastic modulus (126.2 ± 14.6 Pa)[31]. In this ECM model, GBM cell lines tend to exhibit shorter (GBM43, GBM10) or similar migration distances (GBAM1) and higher directionality of movement (ratio of net displacement to total migration distance) when compared to 2D monolayer cultures (S1 Protocol and S1 Fig). Within the ECM model, we incorporated human astrocytes and endothelial cells (ECFCs) with fluorescently labelled GBM cells as represented in Fig 1. Using time-lapse confocal microscopy we followed the migration trajectories of the GBM cell lines: GBM43 (primary GBM; mtp53, CDKN2A deletion), GBM10 (recurrent GBM derived from resection, received prior radiation and salvage chemotherapy; wildtype p53, CDKN2A deletion)[42–44], and the stem-like cell line GBAM1 (neurosphere forming, self-renewal, CD133+, SOX2+, Notch+, GFAP-)[31,45]. The cell trajectories were analyzed to evaluate the effect of astrocytes and ECFCs presence on GBM migratory behavior.

### Astrocytes increase GBM migration in a 3D environment

**Fig 2:**
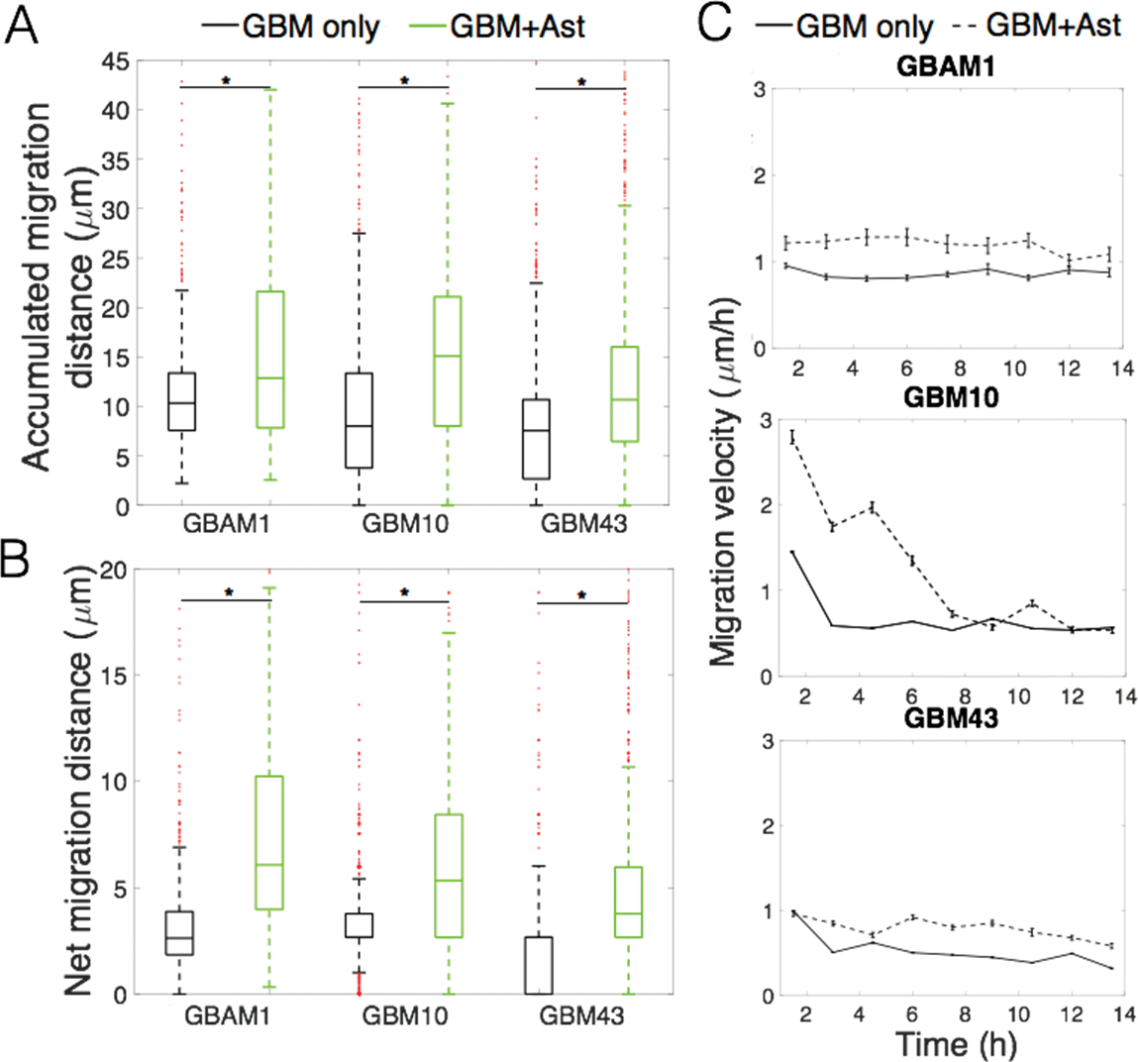
Astrocytes increase the migration GBM cells in a 3D-brain-like model. (A). Accumulated distance of migration, measured over 15 h, increased for all three GBM cell lines. (B). Net displacement between initial (0 h) and final points of migration (15 h) also increased for all cells when co-cultured with human primary astrocytes. (C). The velocity of migration is relatively stable for GBAM1 (CD133^+^) and GBM43 but decreases overtime for GBM10. Data represents a population of 250–2500 individual cells, from at least 2 independent repetitions. Comparison between groups was done by Kruskal-Wallis test. * Represents statistical significant difference at α = 0.05.

To assess the influence of astrocytes on GBM migration in a 3D microenvironment, we incorporated equal numbers of human astrocytes and GBM cells into the 3D collagen-hyaluronan (Col-HA) matrix prior to polymerization. Analysis of the migration patterns indicates that all GBM cell lines studied exhibit higher net displacement (1.9-to 2.4-fold of mean distance, p<0.05) and total distance of migration (1.4- to 1.9-fold of mean distance, p<0.05) when cultured in presence of astrocytes within the 3D matrix (Fig 2A and 2B). Further analysis of the trajectories shows that GBAM1 and GBM43 maintain a relatively constant migration velocity overtime, while the migration velocity of GBM10 reaches a maximum at the beginning of the 3D culture and decreases afterwards (Fig 2C), suggesting a greater initial effect of astrocytes on GBM10 migration that lessens overtime. Similar to 3D results, all GBM cells studied present higher migration distances in 2D in presence of astrocytes (S2 Fig) indicating a consistent effect of astrocytes in both *in vitro* culture models. Interestingly, presence of astrocyte conditioned medium (ACM) increased the migration of GBM10 in 2D, and 3D culture, but its effect on migration was lower than the observed during astrocyte co-culture. GBM43 also presented a slight increase of migration in presence of ACM during 2D culture (S2 Fig), but this effect was not observed in 3D culture (S3 Fig).

### Endothelial colony forming cells (ECFCs) have different effects on migration of GBM cells in a 3D model

**Fig 3:**
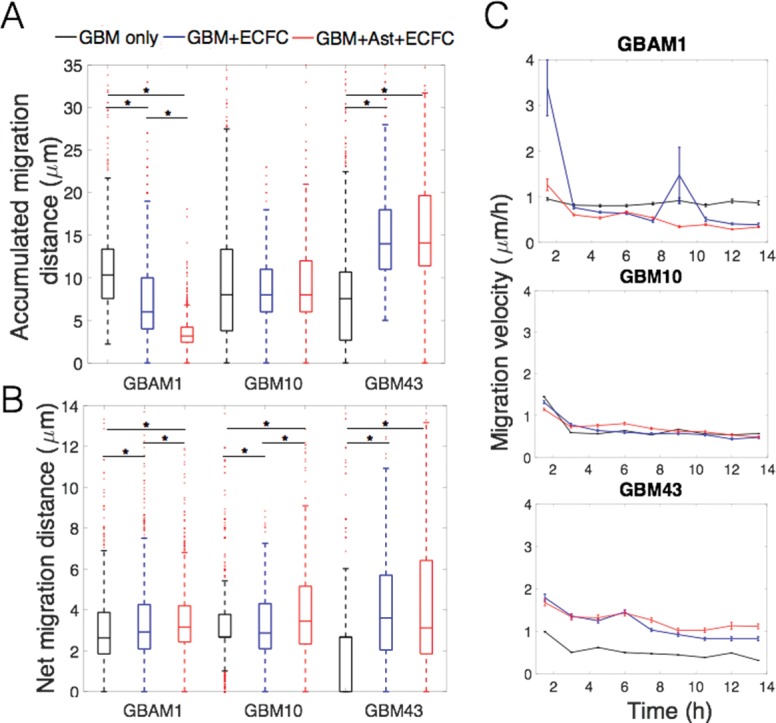
Effect of ECFCs and astrocytes-ECFCs on 3D GBM migration. (A). Accumulated distance of migration over 15 h. (B). Net displacement between initial (0 h) and final points of migration (15 h). Net displacement of GBM cell lines increased due to presence of stromal cells (+ECFC+Ast). (C). The highest migration velocity was observed at the beginning and decreased over time. Migration data represents a population of 250–2500 individual cells from at least 2 independent repetitions for GBM only and from 3 replicates for ECFC and ECFC-Ast. Comparison between groups was done by Kruskal-Wallis test. * Represents statistical significant difference at α = 0.05.

Along with neural and glial cells, endothelial cells help to form the GBM-specific microenvironment. Endothelial precursors, and in some cases stem-like GBM cells, can develop new tumor vasculature and act as migration routes used by GBM to infiltrate healthy brain parenchyma[11,48,49]. Within the 3D Col-HA model, we incorporated endothelial colony-forming cells (ECFCs) alone, and in combination with astrocytes as dual and triple cultures with GBM. GBM43 cells exhibited a significant increase in the accumulated migration distance in presence of ECFC (1.8-fold of the mean distance), and ECFC-astrocytes (2-fold of the mean distance) while GBM10 had a marginal increase in migration (1.1-fold with ECFC and 1.2-fold with ECFC-astrocytes). Presence of ECFCs, alone or in combination with astrocytes, had the opposite effect on the accumulated migration distance of GBAM1 that showed a reduction of by 0.7-fold and 0.6-fold (of mean distance) respectively when compared to control (Fig 3A). ECFCs and astrocyte-ECFCs also increased the net migration of GBM43 but had no effect on the net displacement of GBAM1 (Fig 3B). Despite that GBAM1 mean migration decreased by 0.7-fold in presence of ECFCs compared to control, GBAM1 showed high initial migration velocity that subsequently decreased (similar to GBM10 with astrocytes) which suggests that endothelial cells had a greater initial influence on GBAM1 migration but their effect lessened overtime.

### Presence of stromal cells increases intrinsic migration directionality of stem-like CD133+ GBAM1 cells

**Fig 4:**
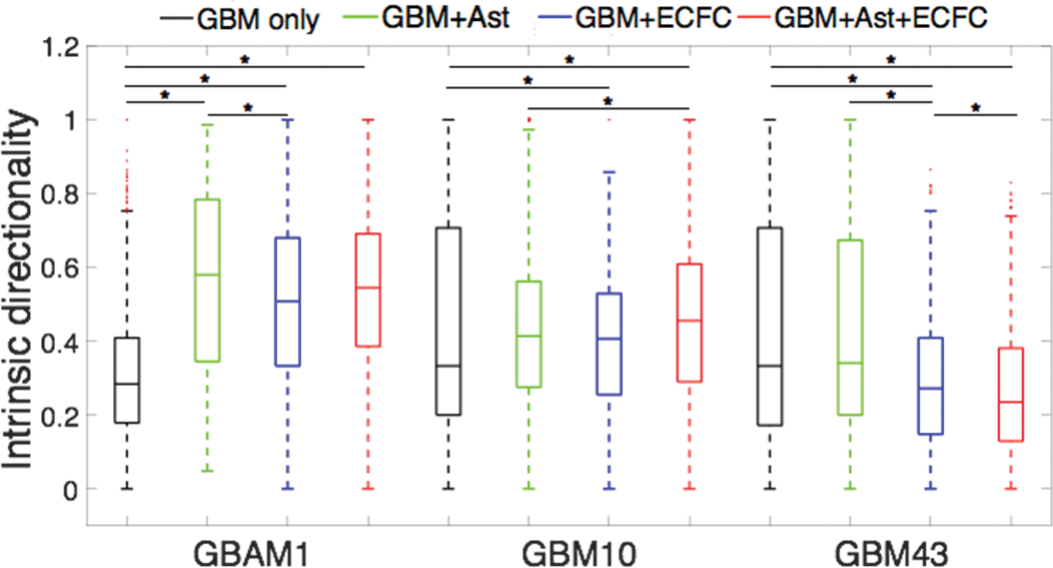
Presence of stromal cells in the tumor microenvironment increases intrinsic migration directionality of GBAM1 (CD133+). Data represents a population of 250–2500 individual cells. GBM only and GBM+Ast correspond to at least 2 independent repetitions per group, GBM+ECFC and GBM+Ast+ECFC correspond to 3 replicates. Comparison between groups was done by Kruskal-Wallis test and Tukey-Kramer mean comparison * Represents statistical significant difference at α = 0.05.

Directionality is a fundamental characteristic of migration, and accounts for the ability of a cell to stabilize the polarization and protrusions required for movement as a response to intrinsic and/or environmental signals[50]. Using the position tracking obtained from time-lapse imaging, we determined the effect of stromal cells in the microenvironment on GBM intrinsic migration directionality–defined as net / accumulated migration—during coculture in the uniform 3D Col-HA matrix. In our case the presence of stromal cells was evaluated as a non-directional (uniform) motogenic signal and no external gradients or guidance cues were applied to the system. The intrinsic directionality of GBM10 and GBM43 cells was slightly reduced in presence of astrocytes, ECFCs, or combination of both cell populations (Fig 4). In contrast, the presence of astrocytes or ECFCs significantly increased the intrinsic migration directionality of GBAM1 (CD133+) by 1.6- to 1.8-fold in all combinations evaluated (Fig 4). The greater intrinsic directionality of GBAM1 in presence of stromal cells might suggest that interactions with stromal cells have a significant effect on polarization and protrusion stability of GBAM1 cells (CD133+) compared to the other cell lines evaluated.

### Presence of ECM modulate basal activity of STAT3 in GBM

Despite the importance of rapid migration of GBM as one of the main causes of treatment failure, few studies have focused on targets that concomitantly affect migration and proliferation. Based on our results in Fig 2 that emphasized the importance of microenvironmental signals (presence of a 3D ECM and stromal cells) as regulators of GBM migration, we targeted STAT3, a direct transducer of TME signals involved in migration and survival pathways.

**Fig 5:**
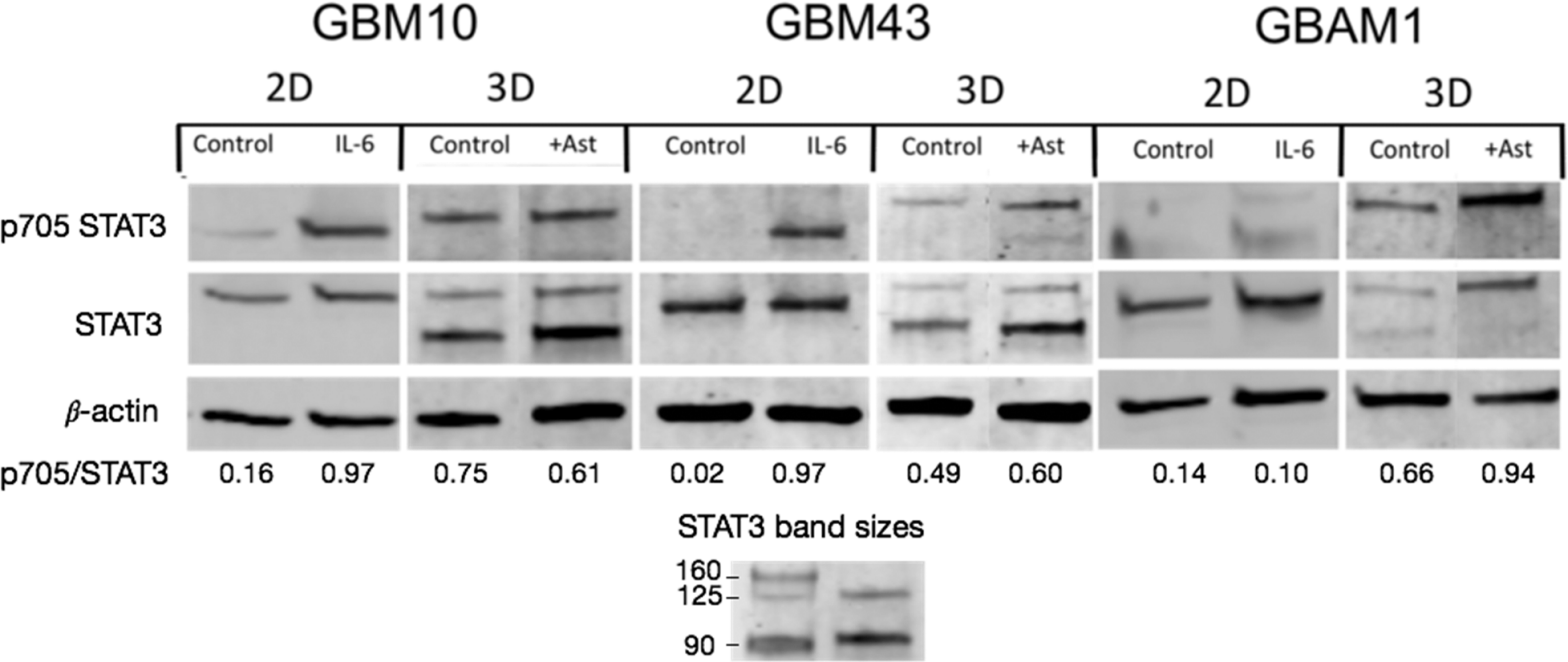
GBM exhibits basal activation of STAT3 when cultured in a 3D matrix that recapitulates characteristics of GBM ECM. Presence of astrocytes in a 3D environment increased the basal STAT3 phosphorylation of GBAM1 compared to 3D culture without astrocytes (see materials and methods for GBM protein recovery during co-culture). STAT3 status of GBM cell lines in 2D culture was tested for at least 2 independent samples with analogous results. Expression of STAT3 from GBM in 3D culture correspond to one sample. Phosphorylation ratio correspond to the images shown.

First, we investigated the impact of including the *in vitro* TME on the basal activation of STAT3 in the tumor cells and compared it with the basal status during 2D monolayer culture. GBM cells displayed very low to absent STAT3 basal activity (evaluated as phosphorylation of Tyr-705) in 2D monolayer culture. Stimulation with interleukin-6 (IL-6), a well-known activator of JAK/STAT signaling, induced a robust but transient phosphorylation of STAT3 in GBM10 and GBM43 but had no effect on the stem-like line GBAM1, in contrast to what has been reported for other stem-like GBM cells[51] (Fig 5). Contrary to 2D culture, all cell lines exhibited basal STAT3 activation when cultured in the 3D GBM-like ECM (Fig 5). GBM10, GBM43, and to a lesser degree GBAM1 exhibited a dual STAT3 band, that we attribute to two different isoforms of STAT3 (possibly, full length STAT3α and C-terminal truncated STAT3β)[52]. Additional presence of astrocytes in the 3D model did not affect STAT3 activation in GBM10 and GBM43, but increased STAT3 activation of the stem-like cell line GBAM1 by nearly 1.5-fold (quantified by image analysis as phosphorylated STAT3 over total STAT3, Fig 5), suggesting a greater effect of the TME on STAT3 activity of GBAM1 compared with the other GBM cell lines. The effect of ECFCs alone or in combination with astrocytes on GBM STAT3 activity could not be evaluated because during co-culture for protein extraction (see Materials and Methods) the matrix containing ECFCs invariably contracted and detached from the GBM-containing matrix.

### Inhibition of STAT3 decreases migration of GBM43 but not GBM10 in 3D brain-like matrix

**Fig 6:**
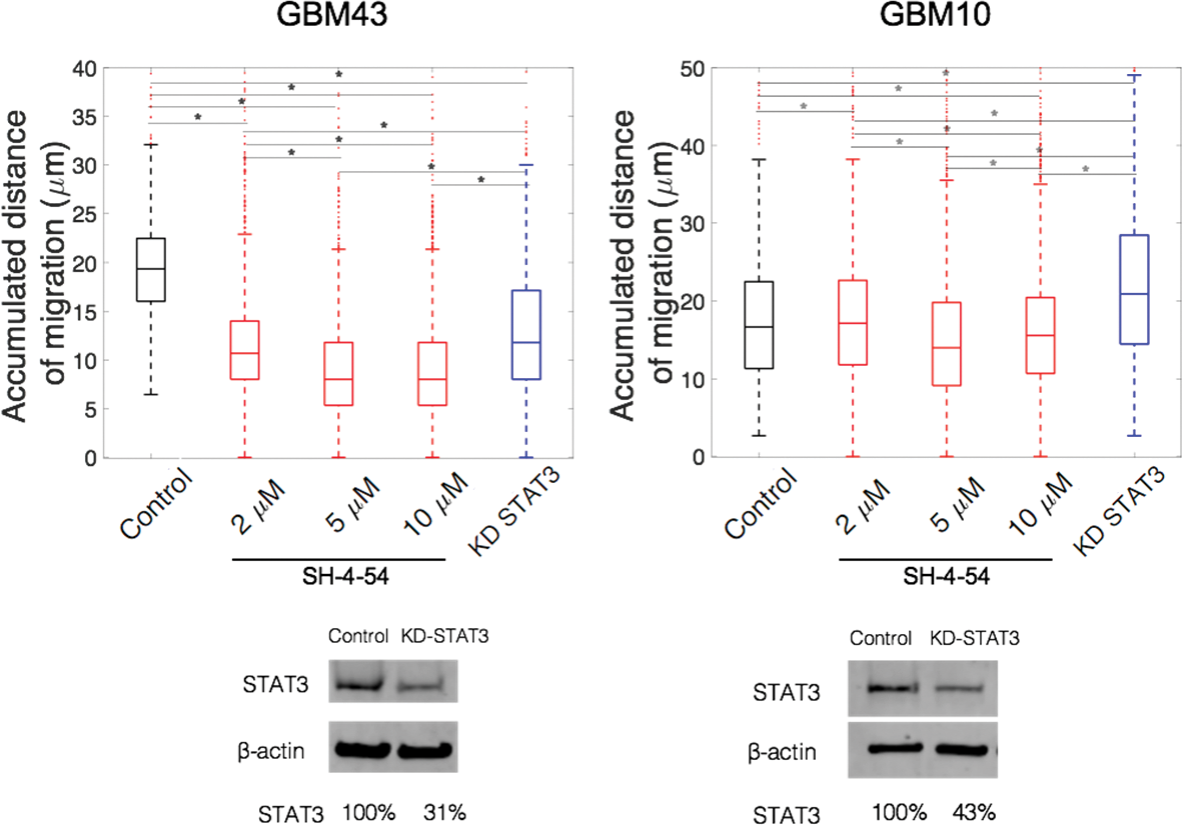
SH-4-54 decreased STAT3 migration of GBM43 but not of GBM10 in a 3D model of GBM microenvironment. GBM43 migration distance decreased with SH-4-54 and STAT3 siRNA knockdown treatments (KD-STAT3) while GBM10 migration was not reduced by the inhibitor SH-4-54 or by STAT3 siRNA treatment. Data represents a population of 250–2500 individual cells from 2 replicates. Comparison between groups was done by Kruskal-Wallis test. * Represents statistical significant difference at α = 0.05. Western blots correspond to cells recovered after 48 h of transfection with STAT3 siRNA or control siRNA A.

Next, we tested the effect of STAT3 drug inhibition on migration of GBM10 and GBM43 using the 3D brain-like matrix, as a possible strategy for targeting GBM migration. To this end, we used both, STAT3 siRNA, and the STAT3 small molecule inhibitor, SH-4-54. SH-4-54 targets the SH2 domain of STAT3 that contains the Tyr-705 residue to block STAT3 phosphorylation and activation [40,41] required for the transcription of genes involved in migration and survival pathways. Dose-dependent treatments with SH-4-54 have been shown to decrease pSTAT3 levels and inhibit expression of genes such as Cyclin D1 and Bcl-xL [40]. First, to confirm the inhibition of STAT3 activation by SH-4-54, we treated cells cultured in 2D with increasing concentrations of SH-4-54 and subsequently stimulated them with IL-6 (S1 Protocol). SH-4-54 did not inhibit STAT3 phosphorylation in GBM10 when treated with 10 μM SH-4-54, however equal drug concentration effectively reduced STAT3 phosphorylation in GBM43 cells to 11% (compared to 100% in stimulated cells with no treatment; S4 Fig), despite both cell lines present wildtype STAT3. GBAM1 cells were not included in these studies due to low basal levels of p-STAT3 and their unresponsiveness to IL-6. We used STAT3 siRNA as well as SH-4-54 to assess the effect of STAT3 signaling on 3D migration. As expected for GBM43 cells, inhibition of STAT3 via treatment with SH-4-54 significantly reduced the accumulated distance of migration of GBM43 (0.5-fold with 10 μM), showing a correlation between increasing drug concentration and reduced migration (Fig 6). STAT3 siRNA treatment also decreased migration distance compared to control cells (scrambled siRNA control). In contrast, no decrease in migration was observed of the recurrent GBM10 line after STAT3 knockdown or following treatment with SH-4-54. STAT3 siRNA treatment increased migration of GBM10 in the 3D environment when compared to control (Fig 6). Such increase may be explained by an incomplete, transient knockdown of STAT3, as the maximum knockdown possible for this line was ~60%. The lack of effect of the SH-4-54 inhibitor on GBM10, in spite of presenting wildtype STAT3, suggests varied effectiveness of this inhibitor, and highlights the heterogeneity of responses that GBM patient-derived cell lines can exhibit after treatment.

### SH-4-54 treatment decreased survival of the stem-like line GBAM1

**Fig 7:**
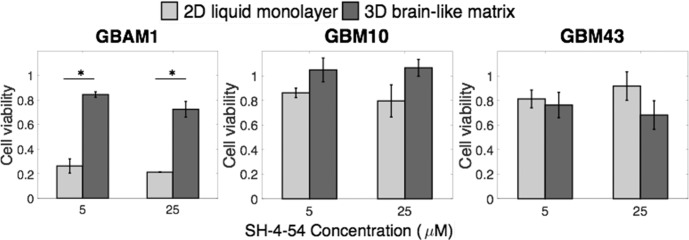
Presence of a 3D brain-like matrix reduces the cytotoxic effect of SH-4-54 in GBAM1 Treatment with SH-4-54 for 72 h drastically reduced cell viability of GBAM1 in 2D liquid culture, but only decreased viability slightly in 3D culture. GBM10 and GBM43 showed a slight decrease in viability after SH-4-54 treatment in 2D culture. The effect of treatment in 2D culture was not significantly different than 3D culture for GBM10 and GBM43. Cell viability was normalized with viability of cells treated with DMSO. Bars indicate Mean ± SE, n> = 3 independent repetitions. Comparison between groups was done by t-test. * Represents statistical significant difference at α = 0.05.

STAT3 activity regulates multiple cell processes including survival and cell proliferation and its inhibition has been previously proven successful to decrease cell viability of GBM initiating cells (also known as stem-like)[40]. Given the different effects of STAT3 inhibition on GBM43 and GBM10 migration, we further investigated the impact of SH-4-54 treatment on GBM survival, both in 2D liquid and 3D culture. In 2D liquid culture, where all cell lines presented low basal STAT3 activity, the inhibitor SH-4-54 had minimal effect on viability of GBM43 and GBM10 cells (Fig 7). In contrast, SH-4-54 effectively reduced viability of the stem-like line GBAM1. Concentrations as low as 5 μM reduced GBAM1 viability to nearly 30%. During 3D culture, the viability of GBM10 was not affected by any of the SH-4-54 concentrations tested, consistent with the lack of reduction in p-STAT3 levels in this cell line (S4 Fig). SH-4-54 treatment slightly decreased viability of GBM43 and GBAM1 in 3D, indicating that STAT3 signaling may play a bigger role in the migration rather than the proliferation of these cells. Given the higher STAT3 activity of GBAM1 in 3D culture (compared to 2D culture, Fig 5), we expected a more dramatic decrease in viability after SH-4-54 treatment, however, we observed reduced effect of SH-4-54 treatment on cell viability upon comparison with 2D culture in GBAM1 cells.

### Presence of stromal cells decreases the effectiveness of SH-4-54 inhibitor on GBAM1 survival

**Fig 8:**
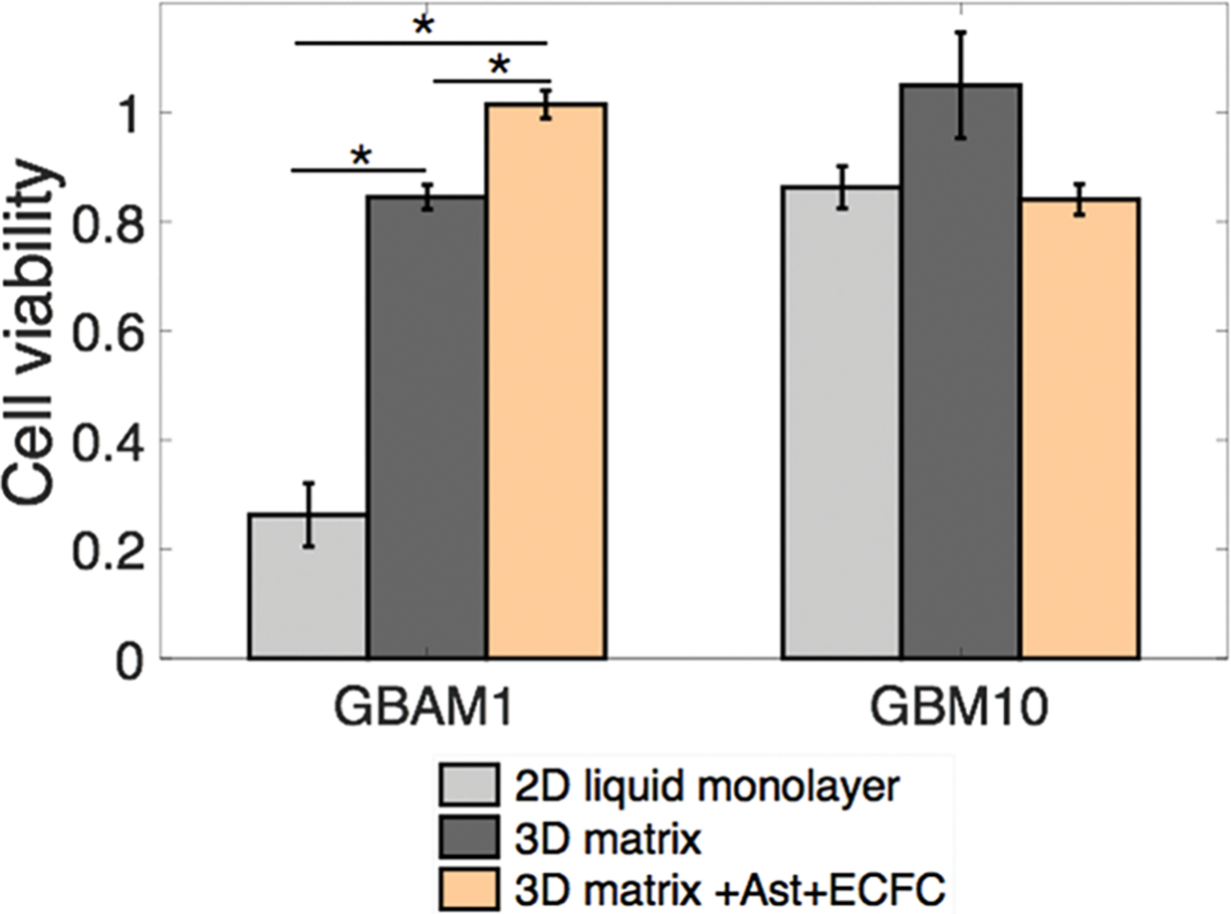
Presence of stromal cells decreased the cytotoxic effect of SH-4-54 on GBAM1 in 3D culture. Astrocytes and ECFCs within the 3D matrix reduced the effect of SH-4-54 (5μM) on GBAM1 viability compared to 3D culture without stromal cells but has no effect on GBM10 viability. Cell viability was normalized with cells treated with DMSO for each of the culture models used. Bars indicate Mean ± SE, n> = 3 independent repetitions. For GBM in co-culture viability analysis was performed as triplicates, each assay with a population of 200–900 individual cells (see materials and methods). Comparison between groups was done by Kruskal-Wallis test and Tukey-Kramer mean comparison * Represents statistical significant difference at α = 0.05.

Based on the effect of stromal cells on STAT3 activity, especially in GBAM1, we further analyzed the effect of SH-4-54 treatment on GBM survival when cultured in presence of stromal cells and the 3D brain-like ECM. Concentration of the inhibitor was set to 5 μM based on the previous results that demonstrated no significant difference on survival compared to a concentration of 25 μM. The presence of astrocytes and ECFCs reduced the effect of SH-4-54 in GBAM1. We observed nearly 100% survival after treatment in presence of stromal cells compared with 80% survival in presence of the ECM and 38% survival in standard 2D liquid culture (without stromal cells) (Fig 8). As a control for SH-4-54 effect, we also tested GBM10. In contrast to GBAM1, GBM10 was resistant to the effects of STAT3 inhibition regardless of the culture conditions. This was expected given the lack of effect of SH-4-54 on STAT3 phosphorylation for this particular cell line.

## Discussion

Despite the fundamental role of ECM and stromal cells as main components of the TME on tumor progression, few studies have incorporated these elements to *in vitro* models to study their effect on glioblastoma behavior. Here we show that the presence of astrocytes within a 3D *in vitro* model of GBM ECM increase GBM migration, while presence of endothelial precursors or astrocytes and endothelial precursors have a varied effect on the migration of various GBM cell lines. Furthermore, presence of stromal cells and a 3D ECM modulates the basal activity of the transcription factor STAT3 and consequently the migration and survival of GBM cells after anti-STAT3 inhibitor treatment.

Stromal cells are fundamental components of the TME and synergistically interact with cancer cells during tumor progression. Specifically in GBM, astrocytes, the main glial components of the brain stroma, have been directly related to increased tumor migration due to astrocytic secretion of matrix metalloproteinases (MMPs)[13], and intercellular communication through gap junctions[53–55].

Previous studies showed that presence of astrocytes or astrocyte conditioned media (ACM) increases the migration of GBM stem-like cells in Transwell assays but has no effect on non-stem GBM cells[32]. Similarly, GBM cells increase migration on nanofibers in presence of rat astrocytes or ACM, yet, present no migration difference in presence of astrocyte-deposited ECM[56]. Consistent with the effect of astrocytes on migration, we observed an increase in the overall migration of both, CD133+, and GBM cell populations -that consist of differentiated cells and small numbers of CD133+ stem-like cells- when astrocytes were incorporated into the 3D matrix. Presence of ACM also increased the migration of GBM10 and GBM43 in 2D culture, but only affected GBM10 in 3D culture suggesting that ACM in presence of ECM can increase migration, as in GBM10, but in other cases, such as GBM43, present no net effect on 3D migration. The lower effect of ACM compared to astrocyte coculture on GBM migration could indicate that soluble factors secreted by the astrocytes can stimulate the migration of GBM but a dynamic and reciprocal interaction between the cell populations further increases the ability of GBM to migrate. Based on the cell density and the homogeneous distribution of both cell populations within the 3D matrix, our results suggest that physical contact between the two populations is not required for an astrocytic effect on 3D GBM migration. It has been previously reported that GBM cells can trigger astrogliosis and cytokine secretion that activate migration[15]. In our 3D platform, it is possible that the effect observed in 3D was mostly due to dynamic signaling between GBM and astrocytes rather than to sporadic physical interactions between cell populations.

Endothelial cells have mainly been studied in GBM in the context of tumor neovascularization. Vascular networks are used by GBM as migration highways, and prolonged inhibition of VEGF can increase GBM migration[57]. *In vitro* models of GBM spheroids have shown that co-culture with endothelial cells increases the expression of genes related to angiogenesis[58]. Here, we used endothelial precursors (ECFCs) able to form vascular network in 3D matrices to simulate the presence of vasculature in the GBM 3D microenvironment. Although endothelial cells present in brain tissue differ from other endothelial cells populations on their ability to tightly regulate transport between brain tissue and blood[59], it is known that GBM compromises the low permeability of brain vasculature[60]. Yet, it is not clear how different endothelial populations interact with GBM *in vitro* and the repercussions in the context of GBM behavior.

Presence of endothelial precursors (ECFCs) and dual presence of ECFCs and astrocytes increased the migration of GBM43 in 3D platforms. However, presence of ECFCs had the opposite effect on GBAM1 and decrease the total migration of this stem-like (CD133+) GBM cell line. Stem-like GBM cells, similar to neural progenitors reside in a specific perivascular niche closely associated with endothelial cells[61,62]. Although, stem-like GBM cells can present high migratory potential under certain conditions, as we and others have shown[31,63], we suggest that components of a perivascular niche, like presence of endothelial cells, provide supportive cues that reduce the migration of stem-like GBM cells. Similarly, the higher intrinsic directionality observed in stem-like GBM in presence of stromal cells can be attributed to intrinsic responses to external guidance cues provided by microenvironment that regulates the cellular polarity machinery and stabilizes the cell leading edge during migration[50]. Incorporation of stromal cells and overall increase of cell density in the 3D model is likely to cause changes in the matrix microstructure and stiffness that affect the ability of cells to migrate[39]. Yet, we did not observe a definite trend relating an increase in cell number with higher GBM migration, suggesting that although physical changes of the matrix might be present, the effect of stromal cells on migration is mainly due to cell-cell specific interactions, especially during short period of times.

Consistent with our results, the TME, and specifically the stromal cells, have a strong impact on GBM that leads to increased migration. Microenvironmental signals are communicated by transcription regulators such as STAT3 that control multiple cellular processes including survival and migration[64,65]. STAT3 is present in the cytoplasm as a non-phosphorylated monomer and undergoes transient phosphorylation and dimerization in response to signals from the microenvironment such as IL-6 family cytokines and certain growth factors[66]. Given its role in multiple oncogenic processes, STAT3 has recently been considered as a possible target to decrease GBM migration and survival. Evaluation of STAT3 basal activity in GBAM1, GBM10 and GBM43 demonstrated that during 2D monolayer culture the cells present low to null constitutive activity (as Tyr-705 phosphorylation), but incorporation of GBM cells into the 3D Col-HA matrix induces STAT3 basal activation.

Changes in the level of STAT3 basal activity due to differences on the physical characteristics of the substrate has also been observed in GBM cells cultured on aligned nanofibers that presented increased STAT3 phosphorylation when compared to cells cultured on flat tissue plates[67]. Presence of the ECM modulates the cell response to soluble signals[68], and increases expression of adhesion molecules, such as β1 integrin, that are known to induce STAT3 activation[69]. Furthermore, increased presence of IL-10 and activity of STAT3 have been directly related to the expression of hyaluronan synthase (HAS2) as well as hyaluronan deposition in the ECM[70,71]. Yet, it is unknown whether the presence of hyaluronan in the ECM can also induce activation of STAT3. Further incorporation of astrocytes into the 3D ECM model increased STAT3 phosphorylation of the stem-like line GBAM1 compared to only 3D ECM. Reactive astrocytes express multiple cytokines and growth factors that directly activate STAT3 including members of the interleukin family IL-6 and IL-10[15,72]. Although, the 3D coculture was modified for protein extraction, and small changes in signal transport could have existed between the two populations, the marked increase of GBAM1 STAT3 activation in presence of astrocytes (nearly 1.5-fold), suggests that GBAM1 shows a greater response to STAT3 activators expressed in presence of astrocytes compared to the other GBM lines studied.

Evaluation of the STAT3 inhibitor, SH-4-54, as a possible treatment to decrease GBM migration, reduced the migration ability of GBM43 in a 3D microenvironment but had no effect on the cell line GBM10 despite both cell lines present wildtype STAT3. Reduction of GBM43 migration following STAT3 inhibition validated previous studies where STAT3 silencing impaired the migration of gastric carcinoma[26] and GBM cells[25,67,73], likely due to decreased activity of RhoA and matrix metalloproteinases (MMPs) expression[74]. The lack of effect of STAT3 inhibition on GBM10 migration suggests STAT3 targeting as a strategy to impair cancer migration effective only for certain patient populations within GBM. STAT3 inhibition has been mainly explored as a way to reduce survival of cancer cells. Constitutive activation of STAT3 directly increases cell survival by upregulation of expression of anti-apoptotic proteins such as Mcl-1, Bcl-2 and Bcl-XL[22,23]. Survival assessment of the various GBM cell lines used in this study, showed that STAT3 inhibition by SH-4-54 was most effective in the stem-like cell line GBAM1 in 2D liquid culture, consistent with previously published work by Haftchenary et al (2013)[40] that shows reduced survival of brain tumor initiating cells after treatment with SH-4-54. SH-4-54 has also been shown to moderately inhibit STAT5 phosphorylation [40]. It is therefore a possibility that STAT5 inhibition might contribute to the reduction of GBAM1 viability in 2D culture. STAT3 inhibition via SH-4-54 minimally decreased the viability of GBM cell line GBM43 and had no effect on GBM10, similar to previous observations of GBM U251 that exhibit no changes on viability after STAT3 inhibition[67]. Indirect reduction of STAT3 by IL-6 targeting has been shown to decrease survival in GBM stem-cells[51]. The effects on cell viability in GBAM1 cells following STAT3 inhibition were drastically reduced when the stem-like cells were grown in 3D and in the presence of the protective TME. This dramatic reduction in treatment effectiveness due to the presence of the TME provides insight into the common failure of drug therapies at early stages during drug development. Similar to our results, others have recognized the effect of the TME on treatment response. GBM stem-like cells cultured on the surface of collagen matrices and treated with multikinase inhibitors presented higher viability after treatment compared to liquid culture[17]. Likewise, presence of stromal cells, such as fibroblasts and astrocytes either in a 2D monolayer co-culture or in heterocellular spheroids have shown to reduce drug effectiveness against breast and brain cancer cells respectively[20,75]. These studies support the use of the TME and 3D matrix interrogated here to determine more effective methods for treating GBM.

## Conclusions

Here, we investigated the effect of the *in vitro* tumor microenvironment (TME) comprised of a 3D ECM and stromal cells (astrocytes and endothelial cells) on the migration and response to STAT3 inhibition in various GBM cell lines. Migration analysis of GBM within a 3D *in vitro* tumor model showed that presence of astrocytes increases the migration of all GBM cell lines studied (GBM43, GBM10 and CD133+ GBAM1) while presence of ECFCs or ECFCs and astrocytes increases the total migration of GBM43 but not the total migration of GBAM1 (CD133+). Moreover, presence of stromal cells increases the intrinsic directionality of migration the stem-like line GBAM1 (CD133+) but have the opposite effect on more differentiated GBM cell lines GBM43 and GBM10. Given the direct regulation of TME signals on regulators of migration and survival such as STAT3, we tested the effect of TME of GBM STAT3 status and observed that both, presence of a 3D ECM, and presence of stromal cells increase STAT3 basal activity in all GBM cell lines studied. Inhibition of STAT3 activity by the small molecule SH-4-54 decreased migration of GBM43 but had no effect on GBM10. Evaluation of SH-4-54 treatment on GBM survival showed a drastic reduction of viability in the stem-like line GBAM1, but minimal effects on the more differentiated GBM cell lines GBM43 and GBM10. Further assessment of the effect of TME on the effectiveness of the anti-STAT3 treatment on GBM viability showed that SH-4-54 treatment effect was reduced in the presence of a 3D ECM and stromal cells. Our results support the critical regulatory effects of the TME on GBM behaviors and validates the use of *in vitro* controllable platforms that recapitulate the characteristics of TME as powerful tools for cancer studies.

## Supporting information

S1 Protocol (DOCX)

S1 Fig Comparison of GBM cell migration in 2D liquid culture vs 3D Col-HA matrix. (A). Accumulated distance of individual cell migration during 15 h. (B). Net migration distance between initial (0 h) and final migration point (15 h). (C). GBM cells showed higher intrinsic directionality (accumulated/net distance) when cultured in a 3D Col-HA matrix. Data represent n = 250–1500 individual cells from three replicates (2D) or from at least 2 independent repetitions (3D). Boxes indicate first, second and third quartile and outliers are presented as red dots. * Represents statistical difference at α = 0.05. n> = 3 independent repetitions. Comparison between groups was done by t-test. * Represents statistical significant difference at α = 0.05.
(TIF)

S2 Fig Presence of astrocytes increases 2D GBM cell migration while presence of astrocyte conditioned media (ACM) only increases migration of GBM10 but not GBM43. (A). Accumulated distance of migration during 15 h. (B). Net migration distance between initial (0 h) and final points of migration (15 h). (C). Directionality of migration (net over accumulated distance). Bars indicate Mean ± SE from a population of 250–1500 individual cells from three replicates. Comparison between groups was done by Kruskal-Wallis. * Represents statistical significant difference at α = 0.05.
(TIF)

S3 Fig Astrocytes and astrocyte conditioned media (ACM) increase the migration of GBM10 in a 3D brain-like matrix while only astrocytes increase 3D GBM43 migration. Presence of living astrocytes has a greater effect than ACM on 3D GBM migration. (A). Accumulated distance of migration during 15 h. (B). Net migration distance between initial (0 h) and final points of migration (15 h). (C). Directionality of migration (accumulated over net distance). Bars indicate Mean ± SE from a population of 240–1500 individual cells from at least 2 independent repetitions. Comparison between groups was done by Kruskal-Wallis test. * Represents statistical significant difference at α = 0.05.
(TIF)

S4 Fig Effect of STAT3 inhibitor SH-4-54 on STAT3 Tyr-705 phosphorylation in GBM43 and GBM10. SH-4-54 effectively decreases phosphorylation of STAT3 in the GBM43 cell line but has no effect on STAT3 activity in GBM10. Total protein loaded per lane 7 μg GBM10, 14 μg GBM43.
(TIF)
