# Supplementary material for: Presence of stromal cells in a bioengineered tumor microenvironment alters glioblastoma migration and response to STAT3 inhibition
Source: PLoS One. 2018 Mar 22;13(3):e0194183. doi: 10.1371/journal.pone.0194183 (PMC5863989; doi:10.1371/journal.pone.0194183)
Supplement: S1 Protocol — (DOCX) [file pone.0194183.s001.docx]

SUPPORTING INFORMATION

Presence of Stromal Cells in a Bioengineered Tumor Microenvironment alters Glioblastoma Migration and Response to STAT3 Inhibition

R. Marisol Herrera-Perez^a,d^, Sherry L. Voytik-Harbin^b,c^, Jann N. Sarkaria^e^, Karen E. Pollok,^f,g,h^ Melissa L. Fishel,^f,g,h^ Jenna L. Rickus^a,b,d^*

a. Department of Agricultural and Biological Engineering, College of Engineering, Purdue University, West Lafayette, Indiana, United States of America

b. Weldon School of Biomedical Engineering, College of Engineering, Purdue University, West Lafayette, Indiana, United States of America

c. Department of Basic Medical Sciences, College of Veterinary Medicine, Purdue University, West Lafayette, Indiana, United States of America

d. Physiological Sensing Facility at the Bindley Bioscience Center and the Birck Nanotechnology Center, Purdue University, West Lafayette, Indiana, United States of America

e. Department of Radiation Oncology, Mayo Clinic, Rochester, Minnesota, United States of America

f. Indiana University School of Medicine, Department of Pediatrics, Wells Center for Pediatric Research, Indianapolis, Indiana, United States of America

g. Indiana University School of Medicine, Department of Pharmacology and Toxicology, Indianapolis, Indiana, United States of America

h. Indiana University Simon Cancer Center, Indianapolis, Indiana, United States of America

* Corresponding Author

[rickus@purdue.edu](mailto:rickus@purdue.edu) (JLR)

S1 Supplemental methods

### Migration on 2D surfaces

Cells were tested for viability, stained with CellTracker™ Green CMFDA dye (Life Technologies, Carlsbad, CA) for visualization and plated on glass-bottom well plates at a density of 15000 cell/cm^2^ and fed with appropriate media (regular GBM media, described in materials and methods or astrocyte conditioned media (ACM)). Plates used for GBAM1 cells were coated with 1.5 μg/ml of Poly-L-lysine in water. For migration assays involving co-culture with astrocytes, astrocytes were initially plated at 15000 cell/cm^2^ density and cultured 24 h before the addition of GBM cells to allow attachment to the plate surface. After GBM addition to the co-culture, cells were fed with media containing equal volumes of complete GBM, and astrocytes media, incubated during 4 h for attachment of GBM cells, and placed in an on-stage incubator chamber to perform time-lapse confocal microscopy every hour during 15 h.

### STAT3 status after drug treatment

GBM10 and GBM43 were seeded in 6-well plates and maintained in 2D standard liquid culture for 36 h (~80% confluency) (see materials and methods). Different concentrations of SH-4-54 (2-10 μM) were added to the cultures and 4 h later the cells were stimulated with IL-6 (Cell Signaling Technologies, Danvers, MA) to a final concentration of 30 ng/ml. The cultures were harvested 30 min after IL-6 addition for protein extraction.
