# Supplementary figures and images for: Presence of stromal cells in a bioengineered tumor microenvironment alters glioblastoma migration and response to STAT3 inhibition
Source: PLoS One. 2018 Mar 22;13(3):e0194183. doi: 10.1371/journal.pone.0194183 (PMC5863989; doi:10.1371/journal.pone.0194183)

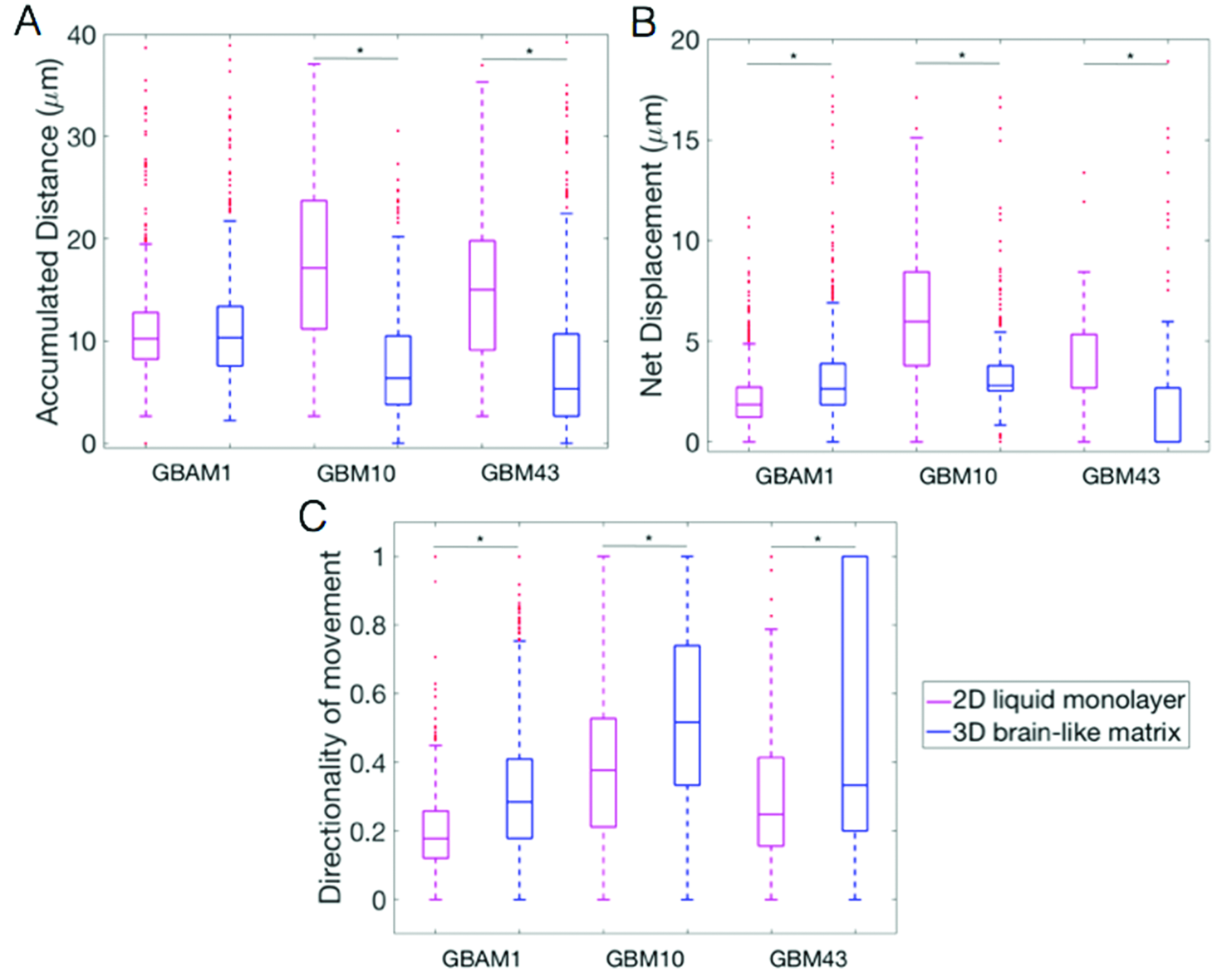

Supplement: S1 Fig — (A). Accumulated distance of individual cell migration during 15 h. (B). Net migration distance between initial (0 h) and final migration point (15 h). (C). GBM cells showed higher intrinsic directionality (accumulated/net distance) when cultured in a 3D Col-HA matrix. Data represent n = 250–1500 individual cells from three replicates (2D) or from at least 2 independent repetitions (3D). Boxes indicate first, second and third quartile and outliers are presented as red dots. * Represents statistical difference at α = 0.05. n> = 3 independent repetitions. Comparison between groups was done by t-test. * Represents statistical significant difference at α = 0.05. (TIF) [file pone.0194183.s002.tif]

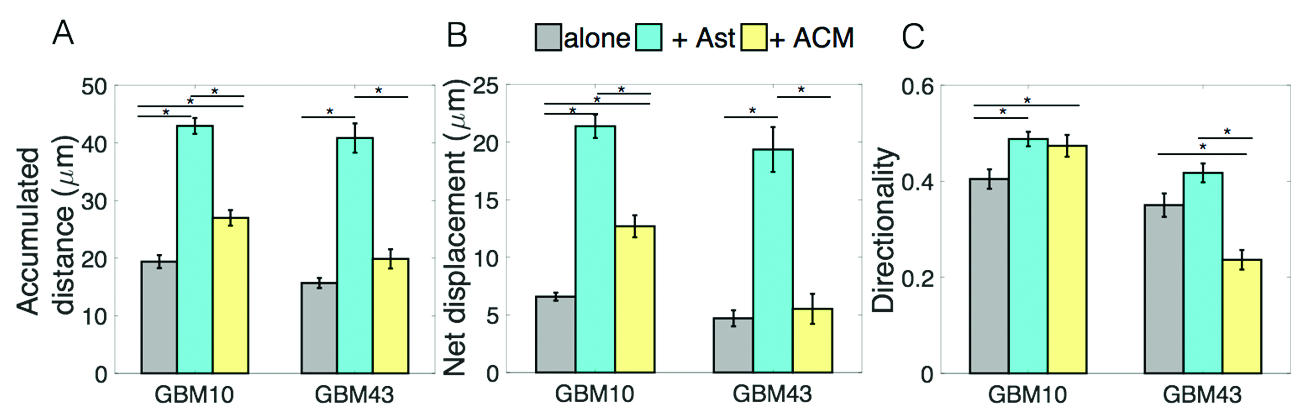

Supplement: S2 Fig — (A). Accumulated distance of migration during 15 h. (B). Net migration distance between initial (0 h) and final points of migration (15 h). (C). Directionality of migration (net over accumulated distance). Bars indicate Mean ± SE from a population of 250–1500 individual cells from three replicates. Comparison between groups was done by Kruskal-Wallis. * Represents statistical significant difference at α = 0.05. (TIF) [file pone.0194183.s003.tif]

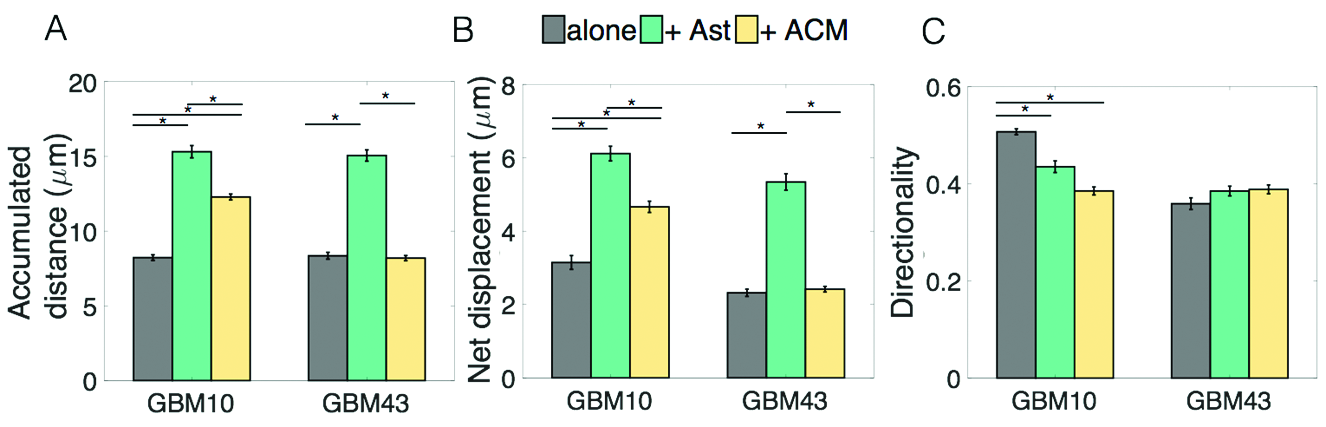

Supplement: S3 Fig — Presence of living astrocytes has a greater effect than ACM on 3D GBM migration. (A). Accumulated distance of migration during 15 h. (B). Net migration distance between initial (0 h) and final points of migration (15 h). (C). Directionality of migration (accumulated over net distance). Bars indicate Mean ± SE from a population of 240–1500 individual cells from at least 2 independent repetitions. Comparison between groups was done by Kruskal-Wallis test. * Represents statistical significant difference at α = 0.05. (TIF) [file pone.0194183.s004.tif]

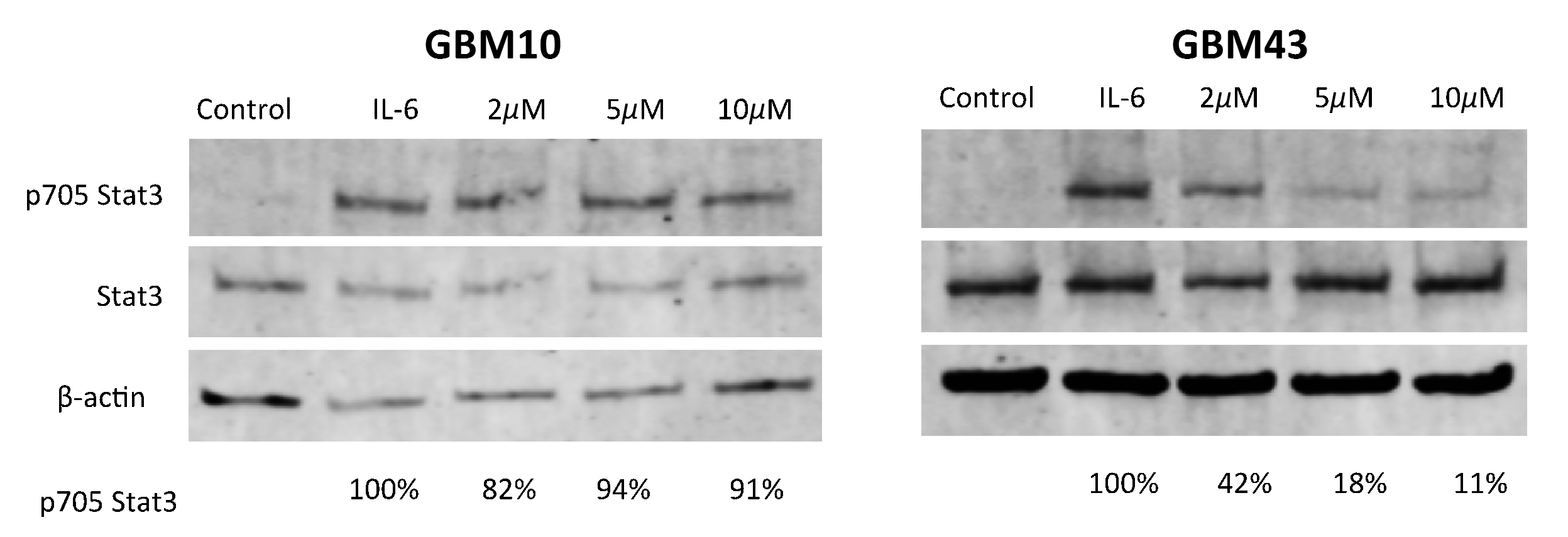

Supplement: S4 Fig — SH-4-54 effectively decreases phosphorylation of STAT3 in the GBM43 cell line but has no effect on STAT3 activity in GBM10. Total protein loaded per lane 7 μg GBM10, 14 μg GBM43. (TIF) [file pone.0194183.s005.tif]
